# Tumour cell PD-L1 expression is prognostic in patients with malignant pleural effusion: the impact of C-reactive protein and immune-checkpoint inhibition

**DOI:** 10.1038/s41598-020-62813-2

**Published:** 2020-04-01

**Authors:** Bahil Ghanim, Anna Rosenmayr, Paul Stockhammer, Melanie Vogl, Ali Celik, Aynur Bas, Ismail Cuneyt Kurul, Nalan Akyurek, Alexander Varga, Till Plönes, Agnes Bankfalvi, Thomas Hager, Martin Schuler, Klaus Hackner, Peter Errhalt, Axel Scheed, Gernot Seebacher, Balazs Hegedus, Elisabeth Stubenberger, Clemens Aigner

**Affiliations:** 1 Karl Landsteiner University of Health Sciences, Department of General and Thoracic Surgery, University Hospital Krems, Krems an der Donau, Austria; 2 Karl Landsteiner Society - Institute for Clinical Surgery, Krems an der Donau, Austria; 3 University Duisburg-Essen, University Medicine Essen – Ruhrlandklinik, Department of Thoracic Surgery, Essen, Germany; 4 Medical University of Vienna, Department of Thoracic Surgery, Vienna, Austria; 5 Gazi University School of Medicine, Department of Thoracic Surgery, Ankara, Turkey; 6 Gazi University School of Medicine, Department of Pathology, Ankara, Turkey; 7 Karl Landsteiner University of Health Sciences, Department of Pathology, University Hospital Krems, Krems an der Donau, Austria; 8 University Duisburg-Essen, University Medicine Essen, Department of Pathology, Essen, Germany; 9 Department of Medical Oncology, West German Cancer Center, University Duisburg-Essen, & German Cancer Consortium (DKTK), Partner site University Hospital Essen, Essen, Germany; 10 Karl Landsteiner University of Health Sciences, Department of Pneumology, University Hospital Krems, Krems an der Donau, Austria

**Keywords:** Lung cancer, Mesothelioma, Metastasis, Tumour immunology

## Abstract

Malignant pleural effusion (MPE) confers dismal prognosis and has limited treatment options. While immune-checkpoint inhibition (ICI) proved clinical efficacy in a variety of malignancies, data on the prognostic role of PD-L1 in MPE is scarce. We retrospectively studied PD-L1 tumour proportion score and Ki-67 index in pleural biopsies or cytologies from 123 patients (69 lung cancer, 25 mesothelioma, and 29 extrathoracic primary malignancies). Additionally, the impact of C-reactive protein (CRP) and platelet count was also analysed. Median overall survival (OS) after MPE diagnosis was 9 months. Patients with PD-L1 positive tumours (≥1%) had significantly shorter OS than patients with negative PD-L1 status (p = 0.031). CRP and Ki-67 index were also prognostic and remained independent prognosticators after multivariate analysis. Interestingly, Ki-67 index and CRP influenced the prognostic power of PD-L1. Finally, patients receiving ICI tended to have a longer median OS and CRP - but not PD-L1 - was a significant prognosticator in this subgroup. In summary, histological and circulating biomarkers should also be taken into account as potential biomarkers in ICI therapy and they may have an impact on the prognostic power of PD-L1. Our findings might help personalizing immune-checkpoint inhibition for patients with MPE and warrant further prospective validation.

## Introduction

Malignant pleural effusion (MPE) is characterized by devastating outcome ranging from 3 to 12 months median overall survival (OS) after diagnosis^1^. MPE is diagnosed by cytology of the pleural fluid and/or by histological examination of pleural biopsy identifying pleural carcinosis. The incidence of MPE is expected to increase in parallel with the general rise in global cancer incidence since about 15% of all cancer patients ultimately develop MPE^2^. Emerging data on prognostic markers including systemic inflammatory related biomarkers (alone and as part of prognostic scores) have been recently published, validated and discussed to better adapt treatment regimens with respect to quality of life and life expectancy in this special cohort of patients with advanced tumours^3–7^.

In recent years, the pivotal role of the immune system in advanced malignant disease was demonstrated and promising clinical results emerged using immune-checkpoint inhibition in a variety of malignancies^8–11^. Thus, the programmed death ligand 1 (PD-L1) pathway as stereotype of an immune modulating target was also analysed in stage IV lung cancer patients affected by MPE^12,13^ and in malignant pleural mesothelioma^14^.

However, the crosstalk between the tumour and the circulating compartments of the immune system in malignant disease in general and the immuno-oncological mechanisms - including the role of PD-L1 tumour expression - associated with dismal outcome in MPE patients in particular are still incompletely understood. In addition, the immune checkpoint inhibition (ICI) still needs to be developed to a more personalized level in oncology^11^. Thus, we aimed to analyse the prognostic role of tumour PD-L1 expression and routinely available pathological and clinical biomarkers including Ki-67 index and circulating C-reactive protein (CRP) levels in patients diagnosed with MPE. Furthermore, we correlated circulating CRP levels with Ki-67 and PD-L1 expression in order to better understand the altered immune mechanisms in advanced oncological patients with pleural dissemination.

## Results

### Association of PD-L1 expression with clinicopathological parameters

In total 123 patients (53 female, 70 male, mean age: 66.8 ± 12.2, range: 25–91 years) were analysed. Ninety-four patients (76.4%) had thoracic primary malignancies (69 lung cancer (56.1%) and 25 mesothelioma (20.3%)), whereas 29 patients (23.6%) had pleural metastases of extrathoracic primary tumours. The major baseline patient characteristics including effusion management and immune-checkpoint therapy are demonstrated in Table 1.

**Table 1:** Patients characteristics of the whole study cohort (n = 123).

		Number (%)
age	≤67 years	59 (48.0)
	>67 years	64 (52.0)
gender	female	53 (43.1)
	male	70 (56.9)
primary	lung	69 (56.1)
	mesothelioma	25 (20.3)
	breast	9 (7.3)
	GIT	7 (5.7)
	UGT	5 (4.1)
	Head & Neck	3 (2.4)
	GYN	3 (2.4)
	CUP	2 (1.6)
extrathoracic metastases	present	51 (41.5)
	absent	72 (58.5)
effusion management	thoracentesis	12 (9.8)
	chest tube	9 (7.3)
	indwelling chest tube	43 (35)
	talc pleurodesis	22 (17.9)
	pericardial drain/puncture	2 (1.6)
	VATS evacuation	35 (28.5)
immune-therapy	none	100 (83.3)
after MPE diagnosis	yes	20 (16.7)
(NA = 3)	- lung	16
	- mesothelioma	2
	- UGT	1
	- Head & Neck	1

Abbreviations: GIT – gastrointestinal malignancy; UGT – urogenital tract malignancy; GYN – gynecological malignancy; CUP – cancer of unknown primary origin; VATS - video assisted thoracic surgery; MPE - malignant pleural effusion.

**Figure 1:**
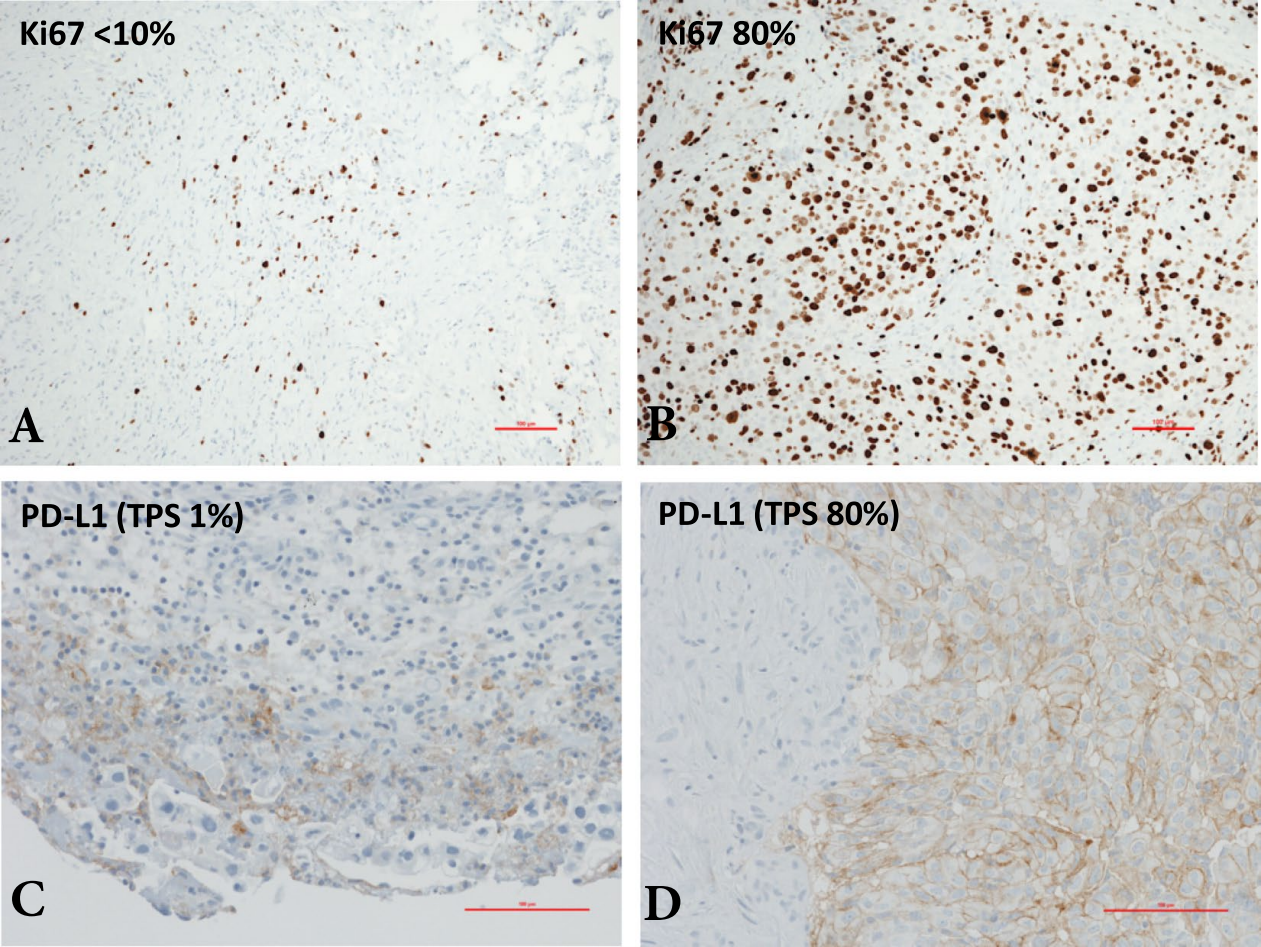
Ki-67 and PD-L1 immunohistochemistry. (**A**,**B**) Malignant pleural mesothelioma specimens with low (<35%) and high (≥35%) Ki-67 index. **(C**,**D)** Low and high tumour cell specific PD-L1 labelling in specimens of lung cancer pleural carcinosis. Scale bars are 100 µm.

When using PD-L1 expression of ≥1% as cut-off, 63 patients (51.2%) were allocated to the PD-L1 negative and 60 patients (48.8%) to the PD-L1 positive group. Among the PD-L1 positive cases, mean percentage of positive tumour cells was 33.6 (±36.1). Figure 1 demonstrates a low PD-L1 expressing tumour sample opposed to a high positive PD-L1 tumour specimen. PD-L1 expression was not significantly associated with the baseline patient characteristics. Only the presence of other extrathoracic metastases (i.e. metastases besides malignant effusion) showed a non-significant tendency towards more PD-L1 positive cases (p = 0.07). No differences where seen in CRP level and in platelet count. All patient characteristics dichotomized by PD-L1 status are demonstrated in Table 2.

**Table 2:** Patients characteristics according to PD-L1 status (n = 123).

Characteristic		PD-L1 positive (≥1% of tumour cells)	PD-L1 negative (<1% of tumour cells)	p-value
mean age in years (±SD)	67.1 (±11.7)	66.6 (±12.8)		0.88*
primary	lung	35	34	NA
	mesothelioma	13	12	
	breast	1	8	
	GIT	1	6	
	UGT	4	1	
	Head & Neck	2	1	
	GYN	2	1	
	CUP	2	0	
gender	female	25	28	0.86^#^
	male	35	35	
extrathoracic	present	30	21	0.07^#^
metastases	absent	30	42	
smoking	never smoker	19	16	0.99^#^
(NA = 26)	ever smoker	35	27	
CRP	<3.8	26	35	0.21^#^
[mg/dL]	≥3.8	34	28	
platelets (±SD) [G/L]		366.1 (±151.9)	330.9 (±125.0)	0.12*
Ki67% positive tumour cells (±SD) (NA = 23)		41.9 (±25.7)	41.5 (±29.0)	0.99*

Abbreviations: GIT – gastrointestinal malignancy; UGT – urogenital tract malignancy; GYN – gynecological malignancy; CUP – cancer of unknown primary origin; CRP - C-reactive protein; ^#^Fisher’s exact test; *Mann Whitney U test.

Furthermore, Ki-67 index was associated with CRP (21 patients with low CRP (<3.8 mg/dl) and high Ki-67 (≥35) vs. 31 patients with high CRP (≥3.8 mg/dl) and high Ki-67, Fisher’s exact test: p < 0.01) but not with the other patient and tumour characteristics (platelet count, PD-L1 positivity, presence of extrathoracic disease and extrathoracic primary tumour). Of note, patients with other metastases besides MPE had a significantly higher mean platelet count when compared to patients with disease limited to the chest (390.9 ± 178.0 G/L vs. 317.6 ± 93.7 G/L, p < 0.01).

### The prognostic impact of PD-L1, Ki-67 index and other patient characteristics

**Figure 2:**
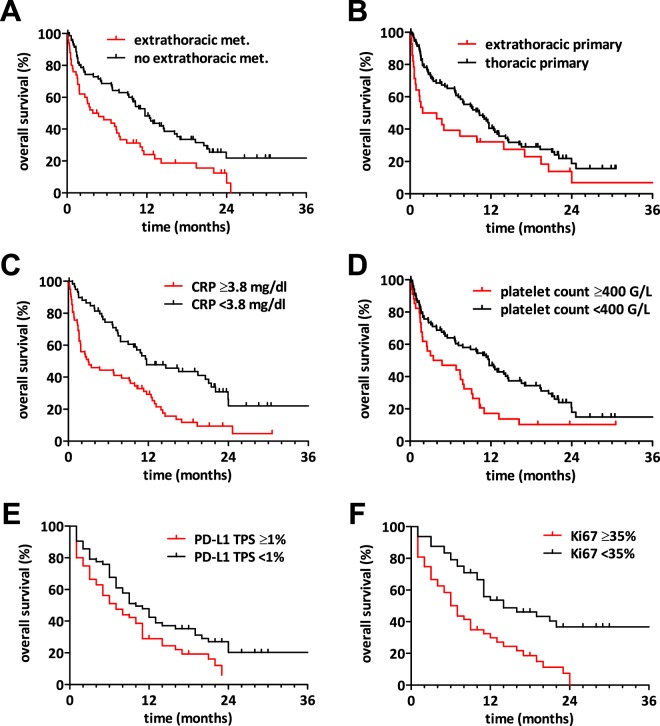
Prognostic factors in patients with MPE. Kaplan-Meier survival curves of MPE patients dichotomized by different clinicopathological characteristics. (**A**) MPE patients with extrathoracic metastases present at the time of MPE diagnosis showed a significantly shorter survival than patients without extrathoracic metastases (HR 1.882, CI 1.246–2.841, p = 0.003). (**B**) MPE patients with extrathoracic primary tumours had significantly worse survival than patients with thoracic primary tumours (HR 1.605, CI 1.007–2.556, p = 0.047). (**C**,**D**) Also CRP levels ≥3.8 mg/dL and elevated platelet counts (≥400 G/L) in MPE patients were significantly associated with shorter survival (CRP: HR 2.288, CI 1.505–3.478, p < 0.001; platelet count: HR 1.877, CI 1.206–2.923, p < 0.005). **(E)** MPE patients with a PD-L1 tumour proportion score (TPS) ≥ 1% had significantly shorter survival than patients with very low or no PD-L1 expression (HR 1.581, CI 1.043–2.396, p = 0.031). **(F)** MPE patients with high Ki-67 index (≥35%) survived significantly shorter than the patient group with low Ki-67 index (HR 2.465, CI 1.513–4.016, p < 0.001).

Median OS after MPE diagnosis was 9 months. Regarding the clinical baseline characteristics, the presence of other extrathoracic metastases at time of diagnosis was associated with worse outcome (hazard ratio (HR) 1.882, 95% confidence interval (CI) 1.246–2.841, p = 0.003, Fig. 2A). On the other hand, extrathoracic primary tumours metastatic to the pleura showed shorter survival time than thoracic primary tumours with pleural involvement (HR 1.605, CI 1.007–2.556, p = 0.047, Fig. 2B).

With regard to the systemic inflammatory parameters, most patients (87.8%) had elevated CRP levels (CRP > 0.5 mg/dl) at time of diagnosis. Furthermore, high CRP (≥3.8 mg/dL) was associated with worse outcome (CRP ≥ 3.8 mg/dL: HR 2.288, CI 1.505–3.478, p < 0.001, Fig. 2C). In addition, elevated platelet count (≥400 G/L) was also significantly associated with worse outcome after MPE diagnosis (platelets ≥400 G/L: HR 1.877, CI 1.206–2.923, p < 0.005, Fig. 2D).

Importantly, PD-L1 tumour expression was identified as prognostic biomarker in patients with MPE. Patients with PD-L1 positive tumours (≥1%) had worse survival when compared to patient with PD-L1 negative (<1%) tumours (HR 1.581 CI 1.043–2.396, p = 0.031, Fig. 2E). Furthermore, PD-L1 tumour expression remained a significant prognostic factor using 50% TPS as cut-off (HR 2.01 CI 1.03–3.90, p = 0.04; Supplementary Fig. 1A). Additionally, we found that the proportion of proliferating tumour cells - reflected by Ki-67 expression in tumour tissue - was a prognostic marker. Accordingly, patients with high Ki-67 tumour expression (≥35%) survived significantly shorter than those with low Ki-67 expression (HR 2.465, CI 1.513–4.016, p < 0.001, Fig. 2F). Of note, Ki67 was prognostic using 14% and 20% cut-offs (p = 0.018 and p < 0.001, respectively). Age (dichotomized by median age, p = 0.841) and gender (p = 0.981) were not significantly associated with overall survival after MPE diagnosis in univariate analyses. Univariate OS analysis is shown in Table 3.

**Table 3:** Prognostic parameters.

characteristic	univariate			multivariate		
	HR	CI	p-value	HR	CI	p-value
extrathoracic metastases present	1.882	1.246–2.841	0.003	2.066	1.229–3.472	0.006
extrathoracic primary	1.605	1.007–2.556	0.047	1.289	0.724–2.262	0.397
CRP ≥ 3.8 mg/mL	2.288	1.505–3.478	<0.001	2.165	1.238–3.788	0.007
platelets ≥400 G/L	1.877	1.206–2.923	0.005	1.305	0.763–2.232	0.330
PD-L1 TPS ≥ 1%	1.581	1.043–2.396	0.031	1.637	1.004–2.674	0.048
Ki67 ≥ 35%	2.465	1.513–4.016	<0.001	1.761	1.036–2.985	0.036

Abbreviations: CI – confidence interval, C-reactive protein – CRP, HR – hazard ratio, TPS – tumour proportion score.

### Multivariate and exploratory subgroup survival analyses

**Figure 3:**
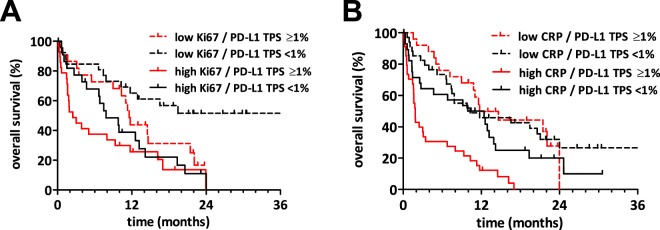
The impact of Ki-67 index and CRP levels on the prognostic power of PD-L1 status. (**A**) Kaplan-Meier survival curves of MPE patients grouped by Ki-67 index (35% cut-off) and PD-L1 tumour proportion score (TPS). In the low Ki-67 index subcohort, PD-L1 positive tumours tended to associate with shorter overall survival (p = 0.075). In PD-L1 negative tumours, Ki-67 index remained a significant prognosticator (p = 0.002), while in the PD-L1 positive cases we identified a strong tendency for shorter overall survival of patients with high Ki-67 index (p = 0.064). (**B**) Kaplan-Meier survival curves of MPE patients grouped by CRP levels (3.8 mg/dL as cut-off) and PD-L1 tumour proportion score (TPS). PD-L1 remained a significant prognostic factor only in the high CRP group (p = 0.003) while completely lost its impact on overall survival in the low CRP group (p = 0.9). CRP was a very strong prognosticator (p < 0.0001) in PD-L1 positive tumours while its prognostic power diminished in PD-L1 negative tumours (p = 0.21).

After showing the univariate prognostic value of PD-L1, we furthermore analysed if the prognostic value of PD-L1 is independent from the other characteristics. In this regard, PD-L1 expression proved to be an independent prognostic marker in our patient cohort (PD-L1 ≥ 1%: HR 1.637 CI 1.004–2.674, p = 0.048). Of note, PD-L1 with 50% as cut-off did not reach statistical significance in the multivariate analysis (p = 0.153, HR 1.603 CI 0.84–3.061). In addition, the presence of extrathoracic metastases, high CRP and high Ki-67 were independent prognosticators associated with poor survival as demonstrated in the right column of Table 3. Interestingly, a significant interaction between CRP and PD-L1 was found in the aforementioned Cox regression model. Accordingly, the corresponding subgroup analyses showed a significant inferior OS of patients with positive PD-L1 tumour status and high CRP as shown in Fig. 3. Besides CRP, no significant interaction was found between PD-L1 and the other characteristics of our multivariate analyses.

**Figure 4:**
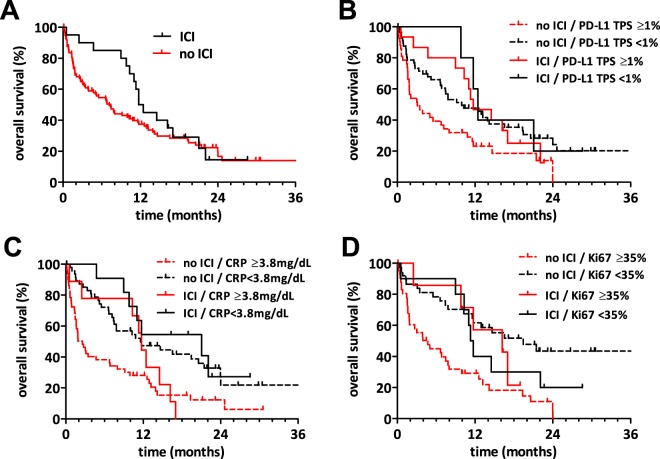
Immune checkpoint inhibitor therapy and overall survival after MPE diagnosis. (**A**) Kaplan-Meier survival curve of MPE patients dichotomized by immune checkpoint inhibition (ICI) treatment after MPE diagnosis showed a trend for shorter survival for patients without subsequent ICI therapy (HR 1.297, CI 0.786–2.139, p = 0.051). (**B**) Kaplan-Meier survival curves of MPE patients grouped by ICI treatment and PD-L1 tumour proportion score (TPS). PD-L1 expression was prognostic in the subgroup of patients without subsequent ICI therapy (HR 1.879, CI 1.145–3.085, p = 0.013). (**C**) Kaplan-Meier survival curves of MPE patients grouped by ICI treatment and CRP levels. CRP levels were prognostic in both subgroups of patients with or without subsequent ICI therapy (no ICI: HR 2.341, CI 1.457–3.762, p = 0.004; ICI: HR 3.044, CI 1.016–9.120, p = 0.047). (**D**) Kaplan-Meier survival curves of MPE patients grouped by ICI treatment and Ki-67 index. Ki-67 expression was prognostic in the subgroup of patients without subsequent ICI therapy (HR 3.168, CI 1.809–5.548, p < 0.0001).

Next, we analysed if patients treated with immune-checkpoint inhibitors (ICI) had a different overall survival (Fig. 4A). Patients receiving ICI treatment had a median overall survival of 12 months compared to 7 months in patients without ICI (Mantel-Cox p = 0.309, Gehan-Breslow-Wilcoxon p = 0.051). In our exploratory subgroup analysis, PD-L1 (1% TPS cut-off), CRP, and Ki-67 remained prognostic in patients without immune-checkpoint inhibition (Fig. 4B–D, p = 0.013, p < 0.0001 and p = 0.0004, respectively). However, PD-L1 and Ki-67 were no significant prognosticators in the subgroup of patients with ICI treatment (p = 0.634 and p = 0.81, respectively). Nevertheless, high CRP remained a significant prognostic factor in the ICI treated subgroup as well (p = 0.047). Survival data for PD-L1 with 50% TPS cut-off and ICI therapy is shown in Supplementary Fig. 1B.

## Discussion

In the current study, we comprehensively analysed the prognostic value of PD-L1 in patients suffering from MPE. For the first time we demonstrated a significant interaction of circulating CRP with tumour cell specific PD-L1 expression indicating that the prognostic value of PD-L1 might be influenced by CRP. In addition, Ki-67 index was an independent prognostic parameter also significantly interacting with ICI therapy in MPE. These results highlight the important prognostic and potentially predictive role of Ki-67 and PD-L1 expression in tumour tissue and of CRP as a circulating systemic inflammation related parameter.

Previously, we demonstrated that an activated innate immune response (reflected by elevated CRP^15^, high fibrinogen^16^, and an altered NLR^17^ and complement system^18^) represents a negative prognostic factor in malignant pleural mesothelioma. Also various other thoracic malignancies including epithelial thymic tumours^19,20^, solitary fibrous tumour of the pleura^21^ and metastatic colorectal carcinoma affecting the lung^22^ showed similar associations. However, the role of the immune system and its related biomarkers beyond the simple prognostic power remains to be elucidated. The underlying biological and immunological mechanisms driving cancer progression and finally leading to poor patient outcome deserve further research.

Interestingly, CRP - as a stereotypic inflammation-related biomarker – proved its independent prognostic power in our cohort of advanced stage patients characterized by pleural dissemination. Similar to previous findings from other groups^7^, we showed that CRP and platelet count were significantly associated with outcome after diagnosis of MPE thus again supporting the theory that an activated innate immune system might translate to poor survival in malignant diseases. In addition, a prognostic value of CRP – as part of a score and alone - in patients receiving ICI therapy was suggested for lung cancer patients before^23,24^.

Besides investigating inflammation related biomarkers, we showed that a high proportion of proliferating tumour cells - reflected by Ki-67 expression - associates with shorter OS in MPE. These findings are in line with our previous study in malignant pleural mesothelioma^17,25^ but also with other studies analysing more prevalent solid tumours like lung^26^ and breast^27^ cancer – both primary tumours that are also represented in our cohort.

Furthermore, Ki-67 tumour expression was significantly correlating with blood CRP levels at diagnosis of MPE suggesting that tumours with high proliferation rate may be more likely to cause an acute phase response (as reflected by CRP). This is in line with our earlier study in MPM^17^. However, these findings are – to the best of our knowledge – novel for pleural carcinosis patients and warrant further validation in prospective studies focusing on distinct malignant diseases.

With regard to the adaptive immune system, its modulation and re-activation by targeting PD-1 or PD-L1 showed promising clinical results in various cancer types including lung cancer^8,10^, which also accounted for most of MPE cases in the present study (56.1%) and in literature^1^. Of note, the presence of MPE in lung cancer patients receiving ICI therapy was described as negative prognostic factor^13^. Furthermore, PD-L1 expression was described as prognostic factor in various solid tumours as summarized in a recent meta-analysis^28^. Nevertheless, very few studies analysed the prognostic impact of PD-L1 in patients with MPE^29^. In line with these findings, we showed that MPE patients with tumour cell specific PD-L1 expression (≥1%) survived significantly shorter than patients with negative PD-L1 status thus suggesting that indeed PD-L1 expression is associated with a more aggressive biological subtype of malignant disease resulting in poor patient survival. This theory is also supported by the fact that multivariate analyses revealed the independent prognostic power of PD-L1 in MPE besides Ki-67 index, extrathoracic metastases and high CRP level at MPE diagnosis.

Most interestingly, the aforementioned prognostic impact of PD-L1 was interacting with CRP potentially suggesting that – besides the suppression of the adaptive immune system – a systemic acute phase response worsens outcome in MPE patients with PD-L1 positive tumours. To the best of our knowledge, this is the first demonstration of this kind of interaction of CRP and PD-L1 expression.

Of note, patients with extrathoracic metastases had significantly higher (p < 0.01) platelet count when compared to patients presenting with disease restricted to the chest only, indicating that platelets might support systemic metastatic processes as previously demonstrated and summarized^30–32^. Concerning CRP and Ki67, we found no association with the presence of extrathoracic metastasis (p = 1.0 and p = 0.44, respectively). Nevertheless, PD-L1 positivity (as defined by ≥1%) tended to associate with extrathoracic metastases (p = 0.07). In patients presenting with MPE the further management will depend on prognosis on the one hand and available appropriate therapeutic options on the other hand^7^.

The roles of tumour or immune cell specific PD-L1 expression as well as the establishment of the clinically relevant cut-offs are still under investigation. Since a number of our specimens were cytology preparations from pleural effusions we could not use immune cell specific or combined scores. With regard to PD-L1, the threshold of 1% and 50% is the most frequently used in recent major lung cancer studies^33–35^. In our study, the 1% cut-off was a significant prognosticator in both, univariate and multivariate analysis. The 50% threshold for PD-L1 was prognostic in univariate but not in multivariate analyses. One explanation might be the relatively low number of patients with >50% PD-L1 expression and with complete data for multivariate analyses (n = 16).

One important limitation of our study is that due to the limited amount of material available for analysis the issue of intratumoural heterogeneity regarding PD-L1 expression cannot be addressed. Recent studies suggest that one small biopsy sample might not be sufficient to represent the tumours PD-L1 status in primary lung cancer^36–38^.

Finally, the retrospective nature of the study results in inherent limitations. ICI was administered to the patient according to the physician´s choice which was probably influenced by the clinical status of the patient. Furthermore, the number of patients receiving immune-checkpoint inhibitors after MPE diagnosis was relatively low (n = 20). Thus, the treatment efficacy of ICI therapy in MPE and the hypothesis that the negative prognostic effect of a positive PD-L1 status or high Ki-67 index is influenced by therapy can only be validated in a prospective setting. Nevertheless, our retrospective multicentre study gives first hints and can serve as basis for follow-up studies to validate our novel retrospective observations.

In summary, we showed for the first time the independent prognostic role of both Ki-67 index and tumour cell specific PD-L1 expression in patients with MPE. Furthermore, we found that CRP, Ki-67 index and PD-L1 expression might impact ICI therapy outcome warranting further investigation as potential predictive biomarkers.

## Methods

### Patients

A retrospective international multicentre analysis was performed after approval of the corresponding local ethic committees. The study was conducted according to the Declaration of Helsinki. All patients suffered from MPE proven either by pleural cytology and/or by biopsy. For PD-L1 staining 32 cytology and 91 biopsy cases were analysed. In Austria, all consecutive 58 patients with complete follow-up and available tumour PD-L1 status at time of MPE diagnosis presenting to the University Hospital Krems from 01/2015 to 09/2017 were included. The 42 patients from Germany were diagnosed between 08/2016 and 05/2018 by the University Medicine Essen – Ruhrlandklinik. Furthermore, 23 patients diagnosed between 04/2013 and 11/2018 were contributed by the Gazi University School of Medicine, Ankara, Turkey.

Cytology specimens were generally obtained from 20 ml pleural fluid, the lowest amount of pleural effusion was 2 ml. For Ki67 and PD-L1 evaluation a minimum of hundred tumour cells were reviewed. All patient samples (either pleural carcinosis or pleural fluid tumour cells) tested during this period were stained by the PD-L1 antibody (clone SP263, Ventana, Roche Diagnostics) and scored by the local Departments of Pathology during routine diagnostic workup. Of note, a recent study demonstrated a high correlation and concordance between histology specimen and matched pleural fluid based PD-L1 evaluation in lung adenocarcinoma^39^. Tumour cell specific PD-L1 staining was provided as percentage of total tumour cells. Patients were dichotomized to the PD-L1 positive and negative group according to 1% PD-L1 positive tumour cells as cut-off. Ki-67 staining and scoring was performed in a similar way by the corresponding local Departments of Pathology. The median percentage of Ki-67 positive tumour cells was 35, which was used for dichotomization into high and low Ki-67 index groups.

With regard to the inflammatory parameters, only 15 patients had – according to the clinical cut-off of 0.5 mg/dl – normal C-reactive protein (CRP) values. Thus, we used the median CRP value of 3.8 mg/dl to dichotomize into high and low CRP groups. With regard to the platelet count the clinical cut-off was used (400 G/L).

Ethical approvals were granted by the corresponding local ethics committees. The Austrian study part was approved by the Kommission für Scientific Integrity und Ethik under the project title “Systemic inflammatory parameters and the programmed death-ligand 1 (PD-L1) pathway in patients suffering from malignant pleural/pericardial effusion – an exploratory single center pilot study”. In Germany the research plan was approved by the ethic committee of the Medizinische Fakultät der Universität Duisburg-Essen under the titles “Prognostische Faktoren bei Lungenkrebspatienten mit malignen Pleuraergüssen (17–7797-BO)” and “Die Rolle von transforming growth factor (TGF)-beta in der Progression des Pleuramesothelioms – eine detailierte Analyse von malignen Pleuraergüssen (17–7773-BO)“. The ethic committee of the Gazi University approved the Turkish part under the project title “Malign plevral/perikardiyal Efüzyonlu Hastalarda Sistemik İnflamatuvar Parametreler ve PD-L1 yolu”. The whole study was performed in accordance with the Declaration of Helsinki. Since this study was based on retrospective analysis of pseudonymized data, the requirement of obtaining informed consent was waived by the Kommission für Scientific Integrity und Ethik of the Karl Landsteiner Private University.

### Statistical analyses

GraphPad Prism 5.0 and IBM SPSS Statistics 24 were used for all statistical analyses. In all analyses, p-values below 0.05 were considered significant. Fisher’s exact test was utilized to identify significant differences between two categorical characteristics. Mann-Whitney test was used to compare metric data between two groups. For all survival analyses, overall survival (OS) is shown. OS is calculated between the day of MPE diagnosis and date of death or date of last follow up (censored cases). Kaplan-Meier survival curves were used to illustrate survival time and demonstrate differences between two or more groups. Log-rank test (unless otherwise stated) was performed to detect significant differences with regard to OS between two or more groups. Furthermore, Cox regression model was used to analyse the impact of different characteristics on patients’ outcome and to calculate the corresponding hazard ratios (HR) and confidence intervals (CI) in uni- and multivariate survival analyses. For multivariate survival analyses, the patient characteristics of univariate impact were included as following: presence of extrathoracic metastases, CRP (dichotomized by median CRP (3.8 mg/dl) level), platelet count (dichotomized by 400 G/L), PD-L1 (dichotomized by one percent positive tumour cells as a clinically often used cut-off as well as closest to the median PD-L1 expression) and Ki-67 tumour expression (dichotomized by the median 35%). For both markers, median values were chosen as thresholds to achieve sufficient patient numbers in the low and high groups for comparison.

For investigating potential interactions between ICI and the other parameters, we added the characteristic ICI therapy after MPE diagnosis to the aforementioned Cox regression model and calculated the corresponding interaction terms between ICI therapy and the other variables. In these types of analyses, we tested PD-L1 at two cut-offs, first at one percent than at 50% positive tumour cells.

## Supplementary information

Supplementary information.

## Data Availability

Upon reasonable request all data and material is available from the corresponding authors.
