# Supplementary material for: Tumour cell PD-L1 expression is prognostic in patients with malignant pleural effusion: the impact of C-reactive protein and immune-checkpoint inhibition
Source: Sci Rep. 2020 Apr 1;10:5784. doi: 10.1038/s41598-020-62813-2 (PMC7113285; doi:10.1038/s41598-020-62813-2)
Supplement: Supplementary file 1 — Supplementary information. [file 41598_2020_62813_MOESM1_ESM.pdf]

Supplementary Information

Tumour cell PD-L1 expression is prognostic in patients with malignant pleural effusion: the impact of C-reactive protein and immune-checkpoint inhibition

Bahil Ghanim, Anna Rosenmayr, Paul Stockhammer, Melanie Vogl, Ali Celik, Aynur Bas, Ismail Cuneyt Kurul, Nalan Akyurek, Alexander Varga, Till Plönes, Agnes Bankfalvi, Thomas Hager, Martin Schuler, Klaus Hackner, Peter Errhalt, Axel Scheed, Gernot Seebacher, Balazs Hegedus, Elisabeth Stubenberger, Clemens Aigner

Supplementary Figure 1.

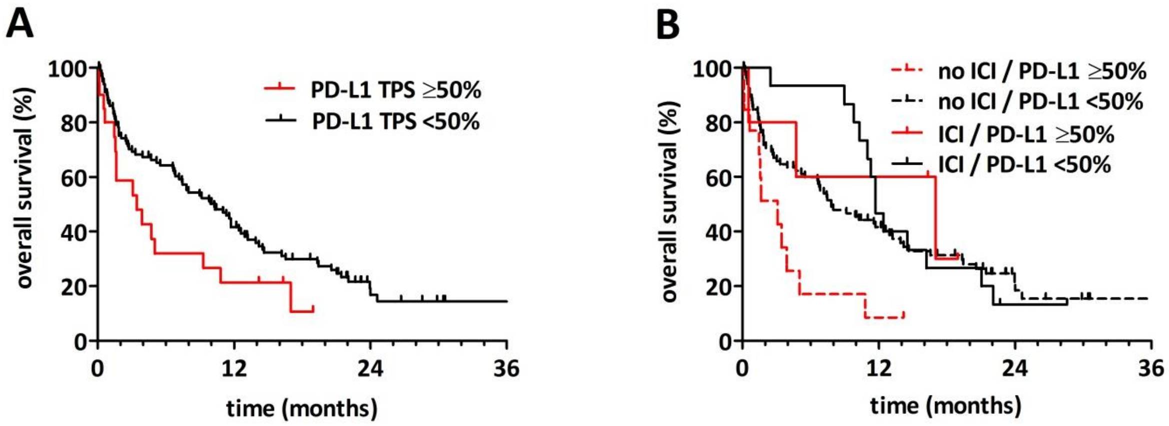

Supplementary Figure 1. Overall survival after MPE diagnosis and 50% PD-L1 tumour proportion score (TPS). (A) Kaplan-Meier survival curve of MPE patients dichotomized by 50% PD-L1 tumour proportion score (TPS). Overall survival was 3.5 versus 10.3 months (HR 2.01 CI 1.03-3.90, p=0.04)**. (B)** Immune checkpoint inhibition (ICI) tended to have a significant impact on overall survival (17 vs 3.1 months; HR 2.92 CI 0.94-9.1, p=0.065) in the subcohort of patients with high PD-L1 expression (TPS≥50%).
